# Facile Synthesis of Bis-Diphenylphosphine Oxide as a Flame Retardant for Epoxy Resins

**DOI:** 10.3390/polym16182635

**Published:** 2024-09-18

**Authors:** Yan Li, Chong Tian, Guiqing Cheng, Chunhui Li, Zhongwei Wang

**Affiliations:** 1 School of Biological and Chemical Engineering, Qingdao Technical College, Qingdao 266555, China; liyan-qd@163.com; 2 College of Materials Science and Engineering, Shandong University of Science and Technology, Qingdao 266590, China; 17864231617@163.com (C.T.); chengguiqing@sdust.edu.cn (G.C.); a17643225062@163.com (C.L.)

**Keywords:** flame retardancy, epoxy resin, (oxybis(4,1-phenylene))bis(phenylphosphine oxide), symmetrical structure

## Abstract

A phosphorus-containing compound, (oxybis(4,1-phenylene))bis(phenylphosphine oxide) (ODDPO), was successfully synthesized and used as a flame retardant for epoxy resin (EP). The results demonstrated that EP/ODDPO, containing 1.2 wt% phosphorus, achieved a vertical burning V-0 rating, with a limited oxygen index value of 29.2%, indicating excellent flame retardancy. Comprehensive evaluations revealed that ODDPO exhibited both gas-phase and condensed-phase flame-retardant effects on EP, with a particularly notable barrier effect. In addition, the incorporation of ODDPO had a minimal negative impact on the glass transition temperature (*T*_g_) and thermal stability of the EP matrix. Compared to unmodified EP (EP-0), the *T*_g_ value and initial decomposition temperature of EP/ODDPO-1.2 decreased by only 7.6 °C and 10.0 °C, respectively. Moreover, the introduction of ODDPO significantly improved the hydrophobicity and water absorption resistance of epoxy materials, which is attributed to ODDPO’s rigidity and symmetric structure, reducing water molecule permeation. Furthermore, the dielectric properties of ODDPO-modified EP samples were strengthened compared to EP-0, due to the ODDPO’s symmetric structure reducing the polarity of the matrix. The above results indicated that ODDPO serves as an excellent flame retardant while enhancing other properties of the EP matrix, thereby contributing to the preparation and application of high-performance epoxy materials.

## 1. Introduction

Epoxy resin (EP), one of the important thermosetting polymer materials, is valued for its excellent mechanical properties, chemical resistance, insulation performance, ease of processing, and thermal stability [1,2,3,4,5,6,7]. It has become essential in modern industry and is widely used in fields such as electronics [8,9,10], coatings [11,12,13,14], adhesives [15,16], construction [17,18], aerospace [19,20,21], and automotive industries [22,23,24,25]. However, EP has the drawback of high flammability, which limits its application in fireproof fields. Therefore, it is necessary and crucial to improve the flame retardancy of epoxy thermosets.

Halogenated flame retardants, widely used to enhance the flame resistance of polymer materials, pose potential risks to the environment and human health due to the release of toxic gases during combustion. Consequently, many industries have banned their use for the sake of environmental concerns. Today, researchers are focused on creating eco-friendly halogen-free flame retardants. Phosphorus-based flame retardants, known for their high efficiency, environmental friendliness, and low toxicity, have become the preferred flame-retardant solution in many industries [26]. Among them, 9,10-dihydro-9-oxa-10-phosphaphenanthrene-10-oxide (DOPO) and diphenylphosphine oxide (DPO) have proven suitable for flame-retardant research across various polymers. Nevertheless, DPO offers significant advantages over DOPO due to its structural benefits, including its higher phosphorus content and the absence of P–O bonds [27,28]. This allows for a lower dosage in polymer modification and improved thermal stability in the modified polymers. Thus, DPO is a highly advantageous and environmentally friendly flame retardant. Wei et al. reported that EP/DPO-0.9 (DPO-modified EP with 0.9 wt% phosphorus) reached a vertical burning (UL-94) V-0 rating, with a limiting oxygen index (LOI) value of 30.5%, showing a prominent flame retardancy [27]. However, compared to unmodified EP, the addition of DPO also led to a noticeable decrease in the glass transition temperature (*T*_g_) and thermal stability of the EP matrix. Furthermore, DPO can form hydrogen bonds with water molecules due to the presence of hydrophilic functional groups in its chemical structure, making it prone to moisture absorption and inconvenient to use [29].

To address the moisture absorption of DPO, increasing focus has been placed on studying DPO derivatives that reduce hygroscopicity by incorporating hydrophobic, or larger, more complex, substituents into their molecular structure. This modification decreases the molecule’s polarity and increases steric hindrance, thereby reducing moisture absorption. Zhang et al. synthesized a phosphorus-containing flame retardant, diphenyl-(2,5-dihydroxyphenyl)-phosphine oxide (DPDHPP), by reacting DPO with benzoquinone and incorporating it into EP [30]. With a phosphorus content of 2.11 wt%, the DPDHPP-modified EP achieved a V-0 rating in the UL-94 test. However, the initial decomposition temperature decreased by 29 °C compared to the unmodified EP. Yuan et al. synthesized a phosphorus-nitrogen compound named DPO-PR from DPO, phenol, paraformaldehyde, and dicyandiamide, and investigated its flame-retardant application in epoxy [31]. The EP modified with DPO-PR demonstrated excellent self-extinguishing properties and achieved a V-0 rating. Nevertheless, the initial decomposition temperature of the modified EP was significantly reduced by 54 °C compared to the unmodified EP. In our previous work, we also extensively investigated the synthesis and application of DPO derivatives. Hydroxy(4-hydroxyphenyl)methyl)diphenylphosphine oxide (DPO-H) was synthesized and utilized as a flame retardant in EP [32]. With a phosphorus content of 0.5 wt%, the DPO-H-modified EP achieved a V-0 rating. However, compared to the unmodified sample, the modified EP exhibited a decrease of 31 °C in *T*_g_ and a decrease of 13.7 °C in initial decomposition temperature. Additionally, we explored the application of the commercial flame retardant 1,2-bis(diphenylphosphinoyl)ethane (EDPO) in epoxy [33]. The findings revealed that, at a phosphorus content of 0.9 wt%, the modified EP attained a UL-94 V-0 rating. However, compared to the unmodified EP, the *T*_g_ decreased by 16 °C, and the initial decomposition temperature decreased by 23 °C.

The studies above indicate that most DPO derivatives can address the problem of high hygroscopicity through structural optimization. However, their enhancements in *T*_g_ and thermal stability have been modest. It has been reported that incorporating a rigid skeleton into the molecular chain of fire retardants can significantly enhance the *T*_g_ value of the EP matrix by restricting thermoset chain movement [34]. Additionally, studies in the literature have demonstrated that flame retardants containing a high content of aromatic groups can improve the thermal stability of epoxy matrices [35]. Furthermore, the polarity of additives has been shown to impact the dielectric properties of EP, a critical factor in electronic applications, where higher polarity molecules typically lead to decreased dielectric properties [36,37].

In this work, (oxybis(4,1-phenylene))bis(phenylphosphine oxide) (ODDPO), a novel phosphorus-containing compound with a high content of aromatic groups, symmetrical structure, and specific rigidity, was successfully synthesized and applied for the flame-retardant modification of EP. Comprehensive properties, including *T*_g_, thermal stability, flame retardancy, flame-retardant mechanism, hydrophobicity, water absorption, and dielectric properties, were investigated in detail.

## 2. Materials and Methods

### 2.1. Materials

Diphenyl ether, dichlorophenylphosphine (DCPP), and 4,4′-diaminodiphenyl sulfones (DDS) were purchased from Shanghai Aladdin Bio-Chem Technology Co., Ltd. (Shanghai, China). Aluminum chloride (AlCl_3_) and hydrochloric acid (HCl) were provided by Qingdao Zhengye Reagent Instrument Co., Ltd. (Qingdao, China). The diglycidyl ether of bisphenol A (DGEBA, grade E-44) was supplied by Zhenjiang Danbao Resin Co., Ltd. (Zhenjiang, China). The dichloromethane was obtained from Tianjin Beilian Fine Chemical Co., Ltd. (Tianjin, China).

### 2.2. Synthesis of ODDPO

**Scheme 1:**
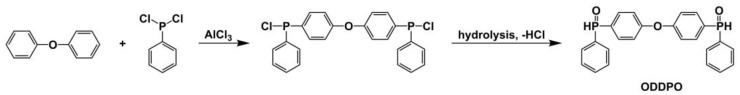
Synthesis route of ODDPO.

The organophosphorus compound ODDPO was synthesized as follows: diphenyl ether (17.1 g, 0.1 mol) and DCPP (46.53 g, 0.26 mol) were mixed in a 250 mL three-necked flask and reacted at 20 °C for 15 h in an N_2_ atmosphere with AlCl_3_ (36.0 g, 0.27 mol) serving as a catalyst. Subsequently, the solution was cooled to room temperature and hydrolyzed with a 10 vol% HCl solution. The resulting mixture was extracted with dichloromethane, and the organic layer was washed with distilled water until neutral. Finally, the organic layer was subjected to vacuum distillation to obtain the target product with a yield of 74.1%. Scheme 1 shows the synthesis route of ODDPO.

### 2.3. Preparation of Epoxy Resin Thermosets

The ODDPO and DGEBA were placed in a 250 mL three-necked flask and stirred under an N_2_ atmosphere at 120 °C until ODDPO completely dissolved. Then, DDS was added to the above solution, and the temperature was raised to 180 °C with continued stirring until DDS dissolved completely. Subsequently, the mixture was subjected to vacuum pumping for 3–5 min to remove air from the solution, thereby preventing the formation of bubbles in the prepared epoxy samples. Finally, the hot solution was poured into the polytetrafluoroethylene mold for curing. Curing conditions were 2 h at 120 °C followed by 2 h at 180 °C. The resulting epoxy samples were labeled EP/ODDPO-x, where x denotes the mass percentage of phosphorus in the modified epoxy. Additionally, an epoxy sample without flame retardants was prepared as a control group under the same conditions and labeled EP-0. Table 1 lists the detailed formulations of epoxy resins with different phosphorus contents.

### 2.4. Characterization

Fourier transform infrared (FTIR) spectra were recorded using a Nicolet 380 infrared spectrometer (Thermo Fisher Scientific, Waltham, MA, USA) over the spectral range from 400 to 4000 cm^−1^.

The structure of the ODDPO was tested by a 400 M Bruker Avance (Bruker, Bremen, Germany) nuclear magnetic resonance (NMR) spectrometer using DMSO-d_6_ as a solvent.

Dynamic mechanical analysis (DMA) was conducted using a DMA1 analyzer (Mettler Toledo, Greifensee, Switzerland) at a frequency of 1 Hz. The specimen with a dimension of 50 mm × 5 mm × 3 mm was heated from room temperature to 250 °C at a rate of 3 °C/min.

Thermogravimetry analysis (TGA) was carried out on an HTG-1 thermogravimetric analyzer (Beijing Hengjiu Experimental Equipment Co., Ltd., Beijing, China). The sample (10–15 mg) was heated at a rate of 10 °C/min from 100 °C to 800 °C under an N_2_ atmosphere.

The LOI value was measured using a BG-5207 oxygen index meter (Suzhou Bengao Instrument Co., Ltd., Suzhou, China) in accordance with the ISO 4589-2017 standard [38], with a specimen size of 130 mm × 6.5 mm × 3 mm. The UL-94 test was conducted on a BG-5210 instrument (Suzhou Bengao Instrument Co., Ltd., China) in accordance with the GB/T 2408-2008 [39], with a specimen size of 130 mm × 13 mm × 3 mm.

The cone calorimeter test was performed on an FTT cone calorimeter (East Grinstead, UK) under an external heat flux of 50 kW/m^2^ according to ISO 5660-1:2015 standard [40], with a specimen size of 100 mm × 100 mm × 3 mm.

The morphology of carbon residue after the cone calorimeter test was observed by Apreo S HiVac scanning electron microscope (SEM, Thermo Fisher Scientific, USA) with an electron voltage of 2 kV. The samples were coated with gold in a vacuum before observation.

Thermogravimetric analysis/infrared spectrometry (TG-IR) was measured using a PerkinElmer 8000/Nicolet IS20 instrument (Shanghai PerkinElmer Enterprise Management Co., Ltd., Shanghai, China). The sample was heated at a rate of 10 °C/min from 50 °C to 800 °C under an N_2_ atmosphere with a flow rate of 100 mL/min.

The static contact angle was measured using a JC2000D1 contact angle meter (Shanghai Zhongchen Digital Technology Equipment Co., Ltd., Shanghai, China) at room temperature. A water droplet of approximately 3 μL was placed on the surface of the sample. After the droplet stabilized, the contact angle was calculated using a five-point fitting method.

The water absorption performance was evaluated through a boiling water experiment. A sample measuring 150 mm × 13 mm × 3 mm was immersed in boiling water. Every 6 h, the sample was taken out, dried, and weighed. The water absorption rate was calculated using the following equation:

(1) $$\mathrm{Water}\;\mathrm{absorption}\;\mathrm{rate}=(\frac{W}{W_{0}}-1)\times 100\%$$

where *W* is the weight of the sample after being placed in boiling water for a certain time, and *W*_0_ is the initial weight of the sample.

Dielectric constant and dielectric loss were measured at frequencies of 1 MHz and 10 MHz at room temperature using an AS2853 digital display high-frequency dielectric measuring instrument (Shanghai Aiyi Electronic Equipment Co., Ltd., Shanghai, China).

## 3. Results and Discussion

### 3.1. Characterization of ODDPO

**Figure 1:**
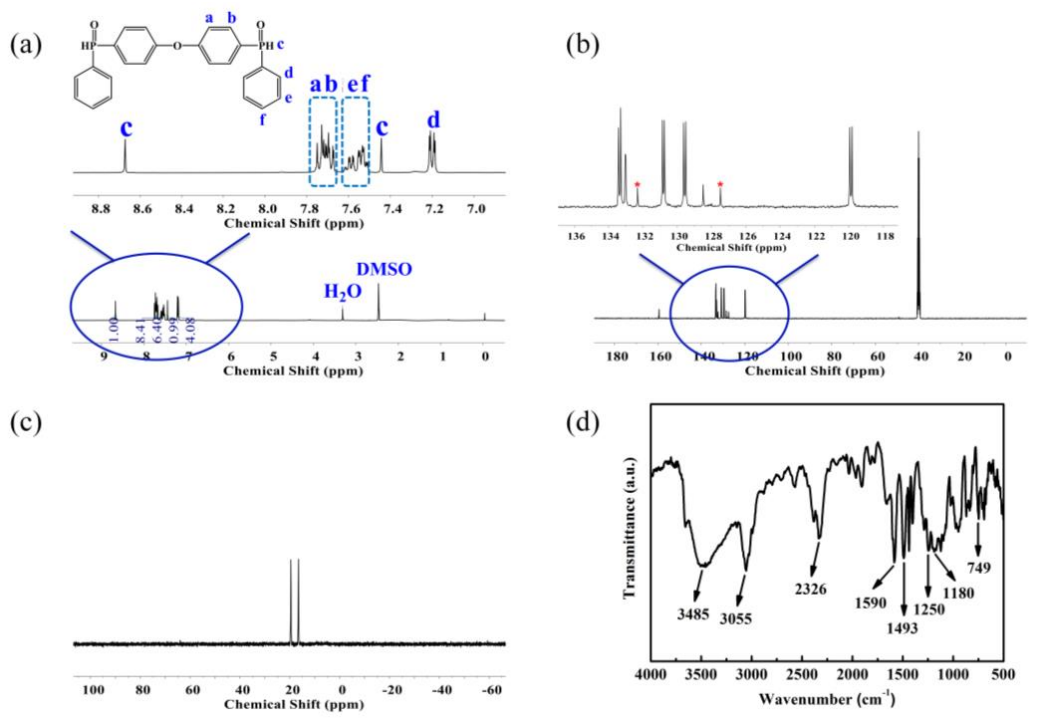
ODDPO characterization: (**a**) ^1^H NMR spectrum; (**b**) ^13^C NMR spectrum; (**c**) ^31^P NMR spectrum; and (**d**) FTIR spectrum. The asterisk (*) indicates impurities in the synthesized compound. The letters are used to represent hydrogen atoms at different positions within the synthesized compound.

The chemical structure of ODDPO was characterized by ^1^H NMR, ^13^C NMR, ^31^P NMR, and FTIR spectra, as shown in Figure 1. In the ^1^H NMR spectrum, the chemical shifts at 8.67 ppm and 7.44 ppm (d, 2H) correspond to the hydrogen peaks at position c of P-H. It should be noted that due to the influence of the phosphorus atom, the hydrogen at position c undergoes coupling, splitting into a doublet with a coupling constant of 491.73 Hz. The chemical shifts at 7.75–7.67 ppm (m, 8H) can be assigned to the hydrogen peaks at positions a and b on the benzene ring. In addition, the chemical shifts at 7.62–7.50 ppm (m, 6H) and 7.22–7.18 ppm (m, 4H) are attributed to the hydrogen peaks at positions e, f, and d on the benzene ring, respectively. In the ^13^C NMR spectrum, eight chemical shifts appeared at 159.52, 133.42, 133.29, 133.00, 130.86, 129.62, 128.48, and 119.94 ppm, corresponding to the eight different carbon atoms on the benzene ring. In the ^31^P NMR spectrum, phosphorus peaks appear at 19.65 ppm and 16.61 ppm. It is reported that phosphinylidene compounds may display prototropic tautomerism [41]. We supposed that isomers of ODDPO are formed during synthesis, showing P(=O)H to P−OH tautomerism. Therefore, ODDPO exhibits two signals in the ^31^P NMR spectrum. The above results confirmed that the synthesized compound is the target product ODDPO.

The product structure was further analyzed by the FTIR spectrum (Figure 1d). The peak at 3485 cm^−1^ corresponds to the stretching vibration of the hydroxyl group [42], which results from P(=O)H to P−OH tautomerism during the synthesis of ODDPO, consistent with the NMR results. The peak at 3055 cm^−1^ was attributed to the C-H stretching vibration on the benzene ring [43]. The peaks at 1590 cm^−1^ and 1493 cm^−1^ were the C-C stretching vibrations on the benzene ring [43]. The P-H stretching vibration can be found at 2326 cm^−1^ [44]. The peaks of P=O, Ar-O-Ar, and P-Ph appeared at 1250 cm^−1^, 1180 cm^−1^, and 749 cm^−1^, respectively [42,45,46]. In summary, the synthesized compound exhibited the structural characteristics of the target product ODDPO.

### 3.2. Thermomechanical Properties of Epoxy Composites

**Figure 2:**
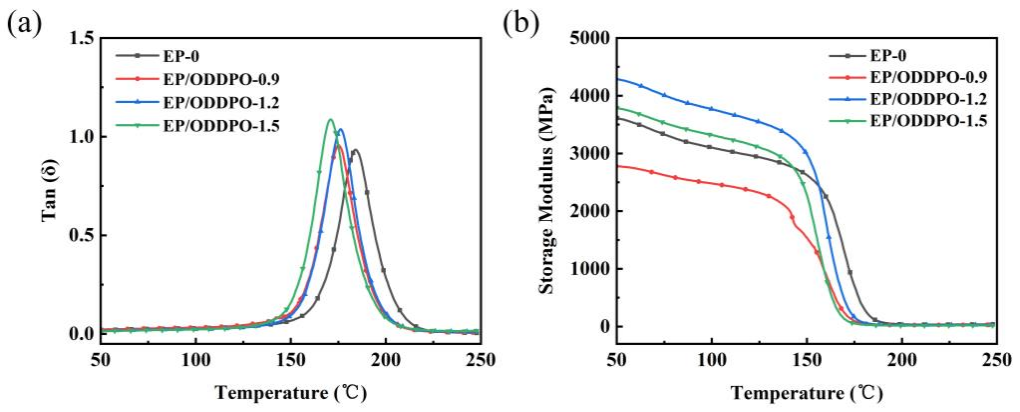
(**a**) DMA; and (**b**) storage modulus of epoxy resins.

DMA was used to test the thermomechanical properties of the cured epoxy samples. The results in Table 2 reveal that the crosslinking density ($v_{e}$) values of ODDPO-modified epoxies are mostly reduced in comparison with the unmodified sample (EP-0). The ODDPO synthesized in this work is a rigid bifunctional flame retardant. The *v*_*e*_ of the EP is influenced by the molecular structure of this flame retardant. At low concentrations, the rigid structure of ODDPO may reduce the mobility of the polymer chains and interfere with their crosslinking, resulting in a decrease in the *v*_*e*_ of EP/ODDPO-0.9 [47]. As the ODDPO content increases, the two ends of the bifunctional additive gradually participate in the polymer’s crosslinking reaction, promoting the formation of new crosslinking points, which leads to a smaller reduction in the *v*_*e*_ of EP/ODDPO-1.2. As a result, the effects of ODDPO’s rigid structure and functional groups on *v*_*e*_ oppose each other. With a further increase in ODDPO concentration, the influence of its rigid structure becomes more dominant than that of the functional groups, leading to a further decrease in the *v*_*e*_ of EP/ODDPO-1.5. As shown in Figure 2a, the *T*_g_ of the ODDPO-modified EP samples was lower than that of EP-0. The *T*_g_ of EP-0 was 184.3 °C, while the *T*_g_ values of EP/ODDPO-0.9 and EP/ODDPO-1.2 were 175.2 °C and 176.7 °C, respectively. The *T*_g_ value is influenced by the $v_{e}$ and the inherent rigidity of the EP thermosets. On the one hand, the rigid structure of ODDPO favored an increase in the *T*_g_ of the matrix with the addition of the flame retardant [48]. On the other hand, a reduction in *v*_*e*_ of the polymer matrix lowers its *T*_g_ value. The combined effects of these factors led to a slight decrease in the *T*_g_ of the epoxy. With a further increase in ODDPO content, the significant decrease in $v_{e}$ led to a reduction in the *T*_g_ of EP/ODDPO-1.5 to 171.5 °C. Additionally, as shown in Figure 2b, the storage modulus (*E*′) of EP/ODDPO-1.2 and EP/ODDPO-1.5 was higher than that of EP-0, possibly because the flame retardant enhanced the rigidity within the epoxy to a certain extent. The $v_{e}$ can be calculated through the *E*′ and *T*_g_ described by Equation (2) [49], as follows:

(2) $$v_{e}=E^{\prime }/3RT$$

where *E*′ is the storage modulus at temperature *T*, *R* is 8.314 J mol^−1^ K^−1^, and *T* is defined as *T_g_* + 40 K.

Although the addition of ODDPO slightly reduced the *T*_g_ of the epoxy matrix, the *T*_g_ value of the ODDPO-modified sample was still higher than that of the epoxy modified by commercial flame retardants (Table 2). Comparative studies reported that the *T_g_* for EP/DOPO-1.2 (DOPO-modified EP with 1.2 wt% phosphorus) and EP/DPO-1.2 (DPO-modified EP with 1.2 wt% phosphorus) were 148 °C and 154 °C, respectively [27]. EP/ODDPO-1.2 exhibited a 19.4% enhancement in *T_g_* compared to EP/DOPO-1.2 and a 14.7% increase compared to EP/DPO-1.2, indicating that ODDPO has a less pronounced effect on the *T*_g_ of the epoxy than both DOPO and DPO.

### 3.3. Thermal Stability of Epoxy Composites

**Figure 3:**
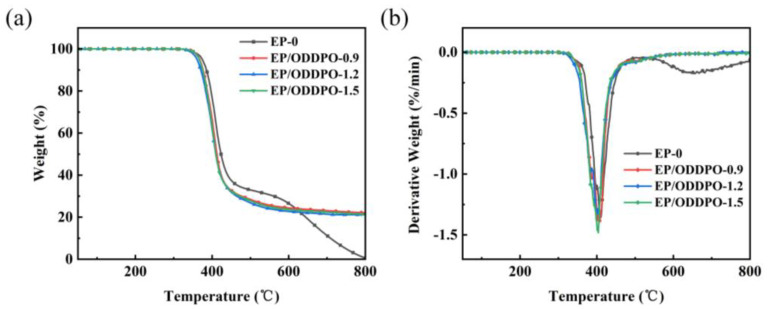
(**a**) TGA; and (**b**) DTG curves of epoxy resins.

The thermal stability of the cured epoxy samples was evaluated using TGA. Figure 3 presents the TGA and DTG curves for the EP-0 and ODDPO-modified EP samples. The char residue at 800 °C, the maximum mass loss rate (MMLR), and the temperatures corresponding to 5 wt% mass loss (*T*_5%_), 10 wt% mass loss (*T*_10%_), and maximum mass loss (*T*_max_) are summarized in Table 2. DTG curves show that all EP samples exhibited a single-stage thermal weight-loss process (Figure 3b), indicating that the introduction of the ODDPO did not alter the thermal degradation behavior of the epoxy matrix. Meanwhile, as the ODDPO content increases, the MMLR of the modified epoxies initially increases, then decreases, and subsequently rises again (Figure 3b and Table 2). This trend may be explained as follows: At low ODDPO content, the flame retardant promotes the decomposition of the EP, leading to rapid weight loss over a short period and resulting in a higher MMLR. At moderate content levels, the flame retardant exhibits a more effective charring effect, slowing down the material’s decomposition and reducing the MMLR. However, at higher content levels, the large amount of volatile products generated during flame-retardant decomposition intensifies the breakdown of the matrix, causing the MMLR to increase once again.

Additionally, compared to EP-0, the characteristic temperatures of EP/ODDPO-0.9 and EP/ODDPO-1.2 gradually decreased (Figure 3a and Table 2). Specifically, the *T*_5%_, *T*_10%_ and *T*_max_ of EP/ODDPO-1.2 were 360.4 °C, 370.5 °C, and 399.2 °C, respectively, which represent decreases of 10.0 °C, 9.1 °C, and 9.1 °C. The stability of the P–C bonds in the flame retardant is lower than that of the C–C bonds in the epoxy matrix. Consequently, the premature decomposition of ODDPO accelerated the breakdown of the epoxy matrix, thereby reducing the thermal stability of the EP samples. It is reported that the *T*_5%_, *T*_10%_*,* and *T*_max_ for EP/DOPO-1.2 were 350 °C, 359 °C, and 374 °C, respectively, while for EP/DPO-1.2 they were 351 °C, 369 °C and 402 °C (Table 2) [27]. These findings suggest that, compared to EP samples modified with DOPO and DPO, ODDPO-modified epoxy exhibited superior thermal stability. With the increase in phosphorus content, the *T*_5%_, *T*_10%_ and *T*_max_ of EP/ODDPO-1.5 decreased to 366.5 °C, 375.1 °C, and 403.9 °C, respectively. This improvement in thermal stability may be attributed to the increased rigidity of the material due to the presence of ODDPO [50]. Furthermore, the char residue of the EP samples increased from 0.7% for EP-0 to 22.6% for EP/ODDPO-1.5, which is beneficial for condensed-phase flame retardancy.

### 3.4. Flame Retardancy of Epoxy Composites

The flame retardancy of the cured EP samples was assessed by the LOI and the UL-94 tests, with the results summarized in Table 3. The EP-0 showed a relatively low LOI value of 23.2% and did not meet the criteria for a UL-94 rating, indicating its inherent flammability. With the addition of ODDPO, the EP/ODDPO-0.9 achieved an enhanced LOI value of 28.1% and reached a V-1 classification in the UL-94 test. Further increasing the phosphorus content in the EP/ODDPO-1.2 and EP/ODDPO-1.5 samples resulted in higher LOI values of 29.2% and 29.9%, respectively, and successful passing of the UL-94 V-0 rating. It is reported that both EP/DPO-1.2 and EP/DOPO-1.2 achieve a UL-94 V-0 rating, with LOI values of 30.8% and 30.0%, respectively (Table 3) [27]. These results demonstrated that ODDPO, as a flame retardant, provides flame retardancy comparable to that of commercial flame retardants. The significant improvement in the flame resistance of EP confirmed ODDPO’s effectiveness as an excellent flame retardant.

### 3.5. Combustion Behavior of Epoxy Composites 

**Figure 4:**
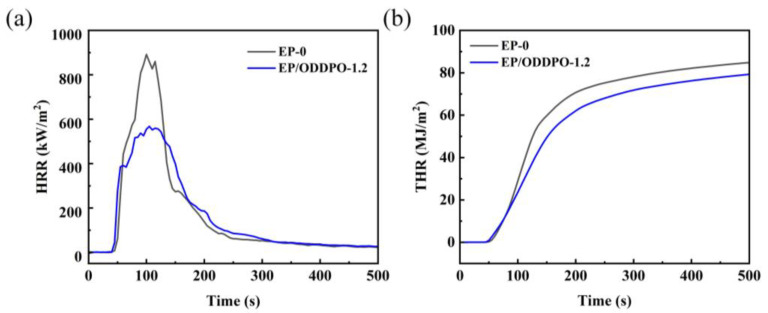
(**a**) HRR; and (**b**) THR curves of epoxy resins.

The combustion behavior of EP samples was evaluated through a cone calorimeter test. The heat release rate (HRR) and total heat release (THR) curves of EP-0 and EP/ODDPO-1.2 are shown in Figure 4, and the detailed relevant data are summarized in Table 4. The time-to-ignition (TTI) for EP-0 was 38 s, whereas the TTI for EP/ODDPO-1.2 was 34 s. This reduction may be due to the premature decomposition of the flame-retardant ODDPO, which promoted the thermal degradation of the epoxy matrix, thereby reducing the TTI. The peak heat release rate (pHRR) for EP-0 was 891.85 kW/m^2^, and THR was 88.20 MJ/m^2^. In comparison, the EP/ODDPO-1.2 exhibited a pHRR of 567.60 kW/m^2^ and a THR of 83.94 MJ/m^2^, ca. 36.4% and 4.8% reduction, respectively. These results indicated that the introduction of ODDPO significantly enhances the flame retardancy of the epoxy matrix.

In general, the average effective heat of combustion (*av-EHC*) can indicate the combustion intensity of volatile substances in the gaseous flame [51]. The *av-EHC* for EP-0 was 22.12 MJ/kg, while it decreased to 20.82 MJ/kg for EP/ODDPO-1.2. This indicated that the introduction of the flame retardant reduces the combustion intensity of volatile gases in the gas phase. The changes in average CO yield (*av-CO*) and average CO_2_ yield (*av-CO*_2_) can also account for this effect. In comparison to EP-0, the *av-CO* of EP/ODDPO-1.2 increased by 20.0%, while the *av-CO*_2_ decreased by 13.1%. The increase in the *av-CO* to *av-CO*_2_ ratio indicated a rise in the incomplete combustion of the EP matrix. These changes may result from the release of phosphorus-containing free radicals during the combustion of the modified EP, which can quench H• and OH• free radicals, interrupting the combustion process and thereby exerting a flame-retardant effect in the gas phase.

Additionally, the char residue increased from 2.42% for EP-0 to 9.16% for EP/ODDPO-1.2, indicating that ODDPO exerts a flame-retardant effect in the condensed phase. This may be attributed to the instability of the P–C bonds in the ODDPO structure, which decomposed upon heating to form polyphosphoric acid, thereby catalyzing the dehydration and charring of the epoxy matrix, consistent with the TGA results.

To understand the flame-retardant mode of ODDPO for EP, its flame-inhibition effect, charring effect, and barrier effect were quantitatively evaluated. The calculations were based on Equations (3), (4) and (5), respectively, with the subscripts FR-EP referring to EP/ODDPO-1.2. Detailed results are summarized in Table 5. From the perspective of gas-phase flame retardancy, the flame-inhibition effect of EP/ODDPO-1.2 improved by 5.88% compared to EP-0. In terms of condensed-phase flame retardancy, the charring effect and barrier effect of EP/ODDPO-1.2 increased by 6.91% and 33.12%, respectively, in comparison with EP-0. This indicated that the barrier effect of the flame retardant is more pronounced, which can prevent or slow down the transfer of heat, oxygen, and flammable gases, effectively inhibiting the combustion process. Overall, the ODDPO exhibited both gas-phase and condensed-phase flame-retardant effects, consistent with the cone calorimeter results.

(3) $$\mathrm{Flame}\;\mathrm{inhibition}\;\mathrm{effect}=1-\frac{{\mathrm{EHC}}_{\mathrm{FR}\text{-}\mathrm{EP}}}{{\mathrm{EHC}}_{\mathrm{EP}\text{-}0}}$$

(4) $$\mathrm{Charring}\;\mathrm{effect}=1-\frac{{\mathrm{TML}}_{\mathrm{FR}\text{-}\mathrm{EP}}}{{\mathrm{TML}}_{\mathrm{EP}\text{-}0}}$$

(5) $$\mathrm{Barrier}\;\mathrm{effect}=1-\frac{{\mathrm{pHRR}}_{\mathrm{FR}\text{-}\mathrm{EP}}/{\mathrm{pHRR}}_{\mathrm{EP}\text{-}0}}{{\mathrm{THR}}_{\mathrm{FR}\text{-}\mathrm{EP}}/{\mathrm{THR}}_{\mathrm{EP}\text{-}0}}$$

### 3.6. Flame Retardancy Mechanism

**Figure 5:**
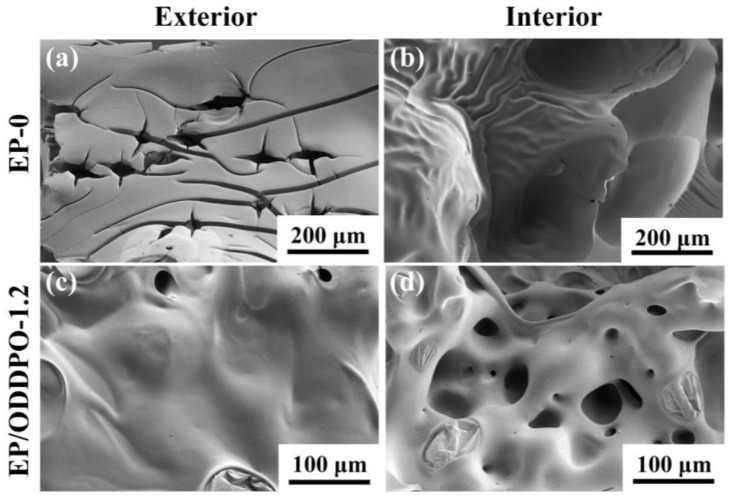
SEM images of the residues for: (**a**,**b**) EP-0; and (**c**,**d**) EP/ODDPO-1.2 after the cone calorimeter test.

The morphology of the char residues of EP-0 and EP/ODDPO-1.2 after cone calorimeter tests was observed using SEM to study the flame-retardant effect of ODDPO in the condensed phase. The external surface of the char residue for EP-0 displayed numerous cracks and holes (Figure 5a), allowing combustible gases and heat to escape, thereby enhancing the combustion of the epoxy matrix. In contrast, the external surface of the char residue for EP/ODDPO-1.2 was compact and continuous, with fewer holes (Figure 5c), effectively acting as a barrier to heat and material exchange. Additionally, the inner surface of the char residue for EP-0 was continuous and without holes (Figure 5b), while that for EP/ODDPO-1.2 exhibited a dense porous structure capable of storing gases produced during the pyrolysis of the matrix (Figure 5d). Once the storage limit is reached, a significant release of these gases will occur, which may help to dissipate heat and reduce the concentrations of oxygen and flammable gases, thereby mitigating combustion [52]. The significant differences in the char structure between the modified and unmodified EP samples indicated that ODDPO plays a role in solid-phase flame retardancy.

**Figure 6:**
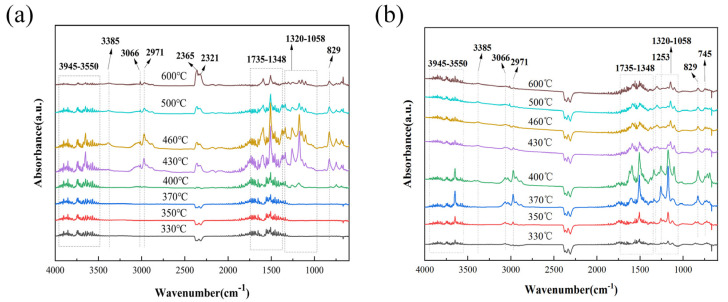
FTIR spectra of gaseous products of: (**a**) EP-0; and (**b**) EP/ODDPO-1.2 at different temperatures.

To further elucidate the gas-phase flame retardancy of ODDPO on EP, the pyrolysis products of EP samples under a nitrogen atmosphere were characterized using TG-IR. Figure 6 presents the FTIR spectra of the pyrolysis products of EP-0 and EP/ODDPO-1.2 at different temperatures. EP-0 decomposed significantly at 430 °C, generating H_2_O (3945–3550 cm^−1^), hydroxyl group (3385 cm^−1^), C-H group of aliphatic hydrocarbons (3066 cm^−1^ and 2971 cm^−1^), CO_2_ (2365 cm^−1^), CO (2321 cm^−1^), aromatic compounds (1735–1348 cm^−1^), compounds containing ethers (1320–1058 cm^−1^), and N-H group from DDS (829 cm^−1^) [42,53,54]. EP/ODDPO-1.2 underwent significant decomposition at 370 °C, attributed to the influence of ODDPO on the thermal stability of the matrix. The gaseous products of EP/ODDPO-1.2 were similar to those of EP-0. Notably, the peak at 1253 cm^−1^ in EP/ODDPO-1.2 was intensified due to the overlap of P=O stretching vibration with the C-O vibration of bisphenol A [45]. Furthermore, EP/ODDPO-1.2 exhibited a new peak at 745 cm^−1^ corresponding to P-C complexes [45]. These two groups consist of phosphorus-containing entities released during the thermal decomposition of the flame-retardant ODDPO. They can combine with H• and OH• free radicals produced from the decomposition of EP, thereby disrupting the combustion reaction, and imparting a gas-phase flame-retardant effect.

### 3.7. Wettability and Water Absorption Properties of Epoxy Composites

**Figure 7:**
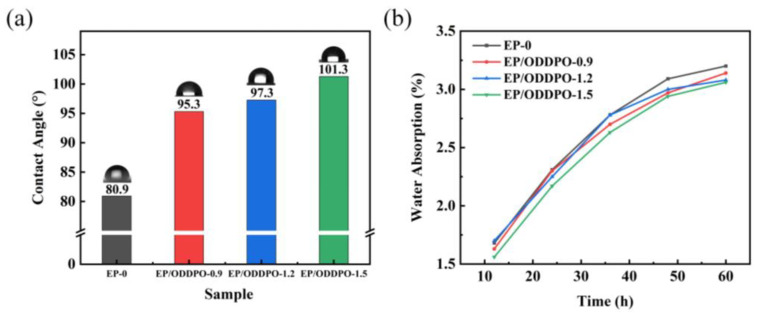
(**a**) Water contact angle; and (**b**) water absorption of epoxy resins.

The hydrophobic properties of EP materials offer unique advantages in many industrial applications, particularly in waterproofing, corrosion resistance, and electrical insulation [55]. It is necessary to study the impact of additives on the wettability of the polymer matrix. Figure 7a shows the water-contact angles of EP-0 and ODDPO-modified EP samples. The water-contact angle of EP-0 was 80.9°, indicating that it is hydrophilic. For EP/ODDPO-0.9, EP/ODDPO-1.2, and EP/ODDPO-1.5, the contact angles were 95.3°, 97.3°, and 101.3°, respectively, showing increases of 17.8%, 20.3%, and 25.2% compared to EP-0. These results indicated that all the ODDPO-modified EP samples are hydrophobic. This is likely due to the symmetric and rigid structure of the flame-retardant ODDPO, which reduced water molecule permeation and enhanced the material’s hydrophobicity [56,57].

The water absorption of EP materials impacts their mechanical properties, electrical insulation, and dimensional stability. Lower water absorption enhances the performance and stability of epoxy in humid environments. Figure 7b shows the water absorption of EP-0 and ODDPO-modified EP samples over time. The water absorption of all samples increased with time. Moreover, the ODDPO-modified EP samples showed lower water absorption compared to EP-0. For instance, the water absorption dropped from 3.20 for EP-0 to 3.06 for EP/ODDPO-1.5 at 60 h. This decrease is likely due to the flame-retardant ODDPO’s symmetric and rigid structure, which reduced the permeation paths for water molecules, thereby lowering the water absorption [56,57].

### 3.8. Dielectric Properties of Epoxy Composites

**Figure 8:**
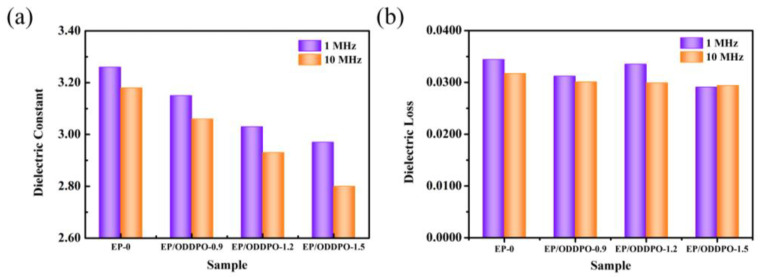
(**a**) Dielectric constant; and (**b**) dielectric loss of epoxy resins.

In electrical applications, a low dielectric constant and a low dielectric loss in insulating epoxy can effectively minimize signal delay and energy loss [58]. Therefore, studying the characteristics of these parameters at different frequencies is crucial for designing and optimizing high-performance electronic devices. Figure 8 illustrates the dielectric constant and dielectric loss of EP-0 and ODDPO-modified EP samples at 1 MHz and 10 MHz. The dielectric constant values for EP-0 at 1 MHz and 10 MHz were 3.26 and 3.18, respectively (Figure 8a). With increases in the flame-retardant content, the dielectric constant of the ODDPO-modified EP samples gradually decreased at both frequencies. For instance, the dielectric constant values of EP/ODDPO-1.5 at 1 MHz and 10 MHz were 2.97 and 2.80, respectively, representing decreases of 8.90% and 11.95% compared to EP-0. Additionally, the dielectric loss of EP-0 was 0.0344 at 1 MHz and 0.0317 at 10 MHz (Figure 8b). As the flame-retardant content increased, the dielectric loss of the ODDPO-modified EP samples also showed a decreasing trend at both frequencies. For instance, the dielectric loss of EP/ODDPO-1.5 was 0.0291 at 1 MHz and 0.0294 at 10 MHz, representing decreases of 15.41% and 7.26%, respectively, compared to EP-0. The modified epoxy samples exhibited lower dielectric constant and dielectric loss, likely due to the presence of ODDPO with its symmetrical structure and small dipole moment, which reduced the polarizability of the epoxy matrix. The above results indicated that the introduction of ODDPO can enhance the dielectric properties of the EP matrix, thereby promoting the application of epoxy materials in electronic and electrical packaging.

## 4. Conclusions

In this study, the successful synthesis of ODDPO and its application as a flame retardant in epoxy thermosets demonstrated significant advancements in flame retardancy while maintaining the material’s intrinsic thermal stability and *T*_g_. With a phosphorus content of 1.2 wt%, the ODDPO-modified EP sample achieved a UL-94 V-0 rating and exhibited a notable increase in the LOI value, to 29.2%. These results, supported by comprehensive cone calorimeter tests, highlight the ability of ODDPO to enhance flame retardancy through both gas-phase and condensed-phase mechanisms, particularly due to its barrier effect, thereby preventing flame propagation. Additionally, the modified epoxies showed improved hydrophobicity, water absorption resistance, and dielectric properties, which could be attributed to the symmetric and rigid structure of ODDPO. These properties make ODDPO-modified epoxies particularly suitable for electronic and electrical packaging applications, where flame resistance, moisture protection, and stable dielectric performance are critical. While the primary focus of this study was on improving flame retardancy and other functional properties, the impact of ODDPO on the mechanical properties of the epoxy composites remains an essential consideration for broader industrial applications. As physical and mechanical properties, such as tensile strength, bending strength, compressive strength, and impact resistance, play a critical role in the selection of materials for structural and load-bearing applications, future research will systematically investigate the effect of ODDPO on these properties.

## Figures and Tables

**Table 1:** The formulations of epoxy resins with different phosphorus contents.

Samples	DGEBA (g)	DDS (g)	ODDPO (g)	P (wt%)
EP-0	100	28.55	0	0
EP/ODDPO-0.9	100	27.34	8.16	0.9
EP/ODDPO-1.2	100	26.91	11.04	1.2
EP/ODDPO-1.5	100	26.47	14.01	1.5

**Table 2:** The DMA and TGA data of epoxy composites.

Samples	*T*_g_ (°C)	*ν_e_* (×10^3^ mol/m^3^)	*T*_5%_ (°C)	*T*_10%_ (°C)	*T*_max_ (°C)	MMLR (%/min)	Char Residue at 800 °C (wt%)	References
EP-0	184.3	5.06	370.4	379.6	408.3	1.36	0.7	This work
EP/ODDPO-0.9	175.2	3.56	367.3	376.1	402.7	1.38	20.4	
EP/ODDPO-1.2	176.7	3.68	360.4	370.5	399.2	1.31	21.5	
EP/ODDPO-1.5	171.5	2.40	366.5	375.1	403.9	1.41	22.6	
EP/DPO-1.2	154	-	351	369	402	-	-	[27]
EP/DOPO-1.2	148	-	350	359	374	-	-	

**Table 3:** LOI values and UL-94 grades of epoxy resins.

Samples	LOI (%)	UL-94 (3 mm)			References
		Dripping	Igniting Cotton	Rating	
EP-0	23.2	Yes	Yes	NR	This work
EP/ODDPO-0.9	28.1	No	No	V-1	
EP/ODDPO-1.2	29.2	No	No	V-0	
EP/ODDPO-1.5	29.9	No	No	V-0	
EP/DPO-1.2	30.8	-	-	V-0	[27]
EP/DOPO-1.2	30.0	-	-	V-0	

**Table 4:** The combustion behavior data of epoxy resins.

Samples	TTI (s)	pHRR (kW/m^2^)	THR (MJ/m^2^)	av-EHC (MJ/kg)	av-CO (MJ/kg)	av-CO_2_ (MJ/kg)	TML (%)	Residue (wt%)
EP-0	38	891.85	88.20	22.12	0.10	1.60	97.58	2.42
EP/ODDPO-1.2	34	567.60	83.94	20.82	0.12	1.39	90.84	9.16

**Table 5:** Quantitative evaluation of the action mode of the flame retardant.

Samples	Flame Inhibition Effect	Charring Effect	Barrier Effect
EP-0	-	-	-
EP/ODDPO-1.2	5.88%	6.91%	33.12%

## Data Availability

The original contributions presented in the study are included in the article, further inquiries can be directed to the corresponding author.
